# Predicting Operational Stability for Organic Light‐Emitting Diodes with Exciplex Cohosts

**DOI:** 10.1002/advs.201802246

**Published:** 2019-02-13

**Authors:** Zhiheng Wang, Mengke Li, Lin Gan, Xinyi Cai, Binbin Li, Dongcheng Chen, Shi‐Jian Su

**Affiliations:** ^1^ State Key Laboratory of Luminescent Materials and Devices and Institute of Polymer Optoelectronic Materials and Devices South China University of Technology Guangzhou 510640 P. R. China

**Keywords:** degradation mechanisms, exciplex cohosts, molecular stability analysis, organic light‐emitting diodes

## Abstract

Organic light‐emitting diodes (OLEDs) employing exciplex cohosts have gained attractive interest due to the promising high efficiency, low driving voltage, and potential low cost in future solid‐state lighting sources and full‐color displays. However, their device lifetime is still the most challenging weakness and rarely studied, which is regarded as a time consuming and complicated work. Therefore, a simplified but effective and comprehensive approach is demonstrated to give prediction for the exciplex cohosts operating lifespan and analyze their possible degradation mechanisms by considering molecular dissociated activation energy with internal exciton dynamics correlations. As a consequence, strong chemical bond stability for the hole transport moieties and rapid reactive exciton relaxation have the intrinsic talent to access potentially long‐lived exciplex cohosts, achieving an extended lifetime of 10169 h for the predicted long‐lived exciplex cohost OLEDs. Degradation behaviors further confirm that the deteriorated source is attributed to the formation of exciton quenchers and hole traps from excited states and charged‐excited states, respectively. The current findings establish a universal technique to screen the stable exciplex cohost candidates with economic time consumption and expenses.

Organic emitters cooperating with appropriate host materials offer a prospective approach to achieve desirable performance in organic light‐emitting diodes (OLEDs). Inside emitting layers, the generated excitons are primarily distributed in host molecules and then transfer energy to organic emitters for efficient light radiation, avoiding aggregation‐caused quenching (ACQ) problems in their emitting dopants. When considering conventional hosts, inherent large singlet–triplet energy split (Δ*E*_ST_) leaves the conflict between wide bandgap to guarantee adequate triplet state and low driving voltage, which is a difficult barrier to be overcome. Furthermore, limited strategies could be achieved to control the carrier balance by modifying transporting moieties or intermolecular interactions.^1^, ^2^, ^3^, ^4^, ^5^ Therefore, to relieve these issues, one of the well‐known solutions is employing an exciplex cohost via intermolecular interaction between electron donor and acceptor molecules, known as hole‐transport material (HTM) and electron‐transport material (ETM). Charge transfer singlet state (^1^CT) and triplet state (^3^CT) possess an intrinsic small Δ*E*_ST_ due to the complete frontier orbital separations.^6^ Therefore, excitation energy of ^1^CT requires much lower energy and low driving voltage is expected. By controlling HTM and ETM ratio, exciplex cohosts show advantages of bipolar transport character and broad carrier recombination region, which eventually facilitates external quantum efficiencies (EQEs) up to ≈25% with favorable efficiency roll‐off control.^7^, ^8^, ^9^, ^10^, ^11^ Several studies have even reported record‐breaking EQEs beyond 30% with horizontal‐orientation emitters.^12^, ^13^, ^14^, ^15^ However, their short operating lifespans still persecute the final commercial application (≈10^6^ h at 1000 cd m^−2^) and are lack of systemic investigation so far.^14^, ^16^

Since actual device fabrication for evaluating exciplex cohost stability is a time consuming and complicated work, the importance to achieve lifetime prediction as well as possible degradation analysis could therefore considerably reduce screening expenses and time consumption. Generally, chemical deterioration of the organic materials and accumulation of the defects in emitting layer (EML) mainly determine the inherent OLED degradation, where most excitons and charged carriers are taken place. Degradation of molecules in charged states, excited states, charged‐excited states, and highly excited states provide the possible mechanisms for molecular destruction. Consequently, in case of employing exciplex cohosts, assessing their molecular stability in the overall degradation mechanisms are essential to screen the potential long‐term candidates. Recent studies have demonstrated that susceptible weak chemical bonds and their possible fragments could be theoretical simulated via identification of dissociated activation energy (*E*_A_) under time dependent density functional theory (TDDFT) calculations.^17^, ^18^, ^19^, ^20^ However, the actual relationship between theoretical molecular stability analysis and their corresponding operating lifespan has not yet been studied, which is the most direct evidence to inspect the theoretical prediction.

The most challenging work to distinguish actual molecular stability is susceptible by the exciton kinetic behaviors in a real electroluminescent device. For instance, device working lifetime shows remarkable development when there is a quick radiative transition to ground state (e.g., high radiative rate, *k*_r_) or energy transfer from high energy state to the lower energy state due to the long triplet lifetime,^21^, ^22^, ^23^ suggesting a proper reactive exciton (exciton in highly excited state) relaxation plays an important role on passivating possible deterioration routes and resolving intrinsic instability. The critical contribution of reactive exciton dynamics should be taken into account within the *E*_A_ descriptor and dissociation rate eventually. Considering the case of exciplex cohosts, their excited state energies are much higher than that of guest emitters and the recombined excitons would distribute on charge transfer states primarily. Hence, the remaining excitons on charge transfer states (^1^CT and ^3^CT) are therefore more susceptible and act as reactive state sources actually. In the purpose of more accurate prediction for exciplex cohost system, it is necessary to coordinate the *E*_A_ descriptor with charge transfer state exciton dynamics and analyze the most possible degradation patterns.

To achieve such challenging conception, we established a new procedure to integrate dissociated activation energy and exciton dynamics behaviors together, predicting molecular stability and dominated degradation mechanism of the involved exciplex cohost candidates. In consequence, molecular stability analysis clearly suggests that rapid reactive state relaxation via Dexter energy transfer, as well as steady chemical bond for the hole transport groups are the intrinsic ability to passivate material deterioration. In this case, the fabricated OLEDs with the predicted long‐lived 9,9'‐diphenyl‐9*H*,9'*H*‐3,3'‐bicarbazole (BCzPh):2‐(9,9'‐spirobi[fluoren]‐2‐yl)‐4,6‐diphenyl‐1,3Δ,5‐triazine (SF2‐TRZ) exciplex cohost successfully realized LT50 (time to 50% of initial luminance at 1000 cd m^−2^) lifetime up to 10 169 h, ≈16 times longer than the reference group tris(4‐(9*H*‐carbazol‐9‐yl)phenyl)amine (TCTA):2,4,6‐tri [(1,1'‐biphenyl)‐3‐yl]‐1,3,5‐triazine (T2T), revealing a corresponding agreement with our molecular stability prediction. Accumulation of luminous quenchers and hole traps in the degraded devices was observed by transient electroluminescence (TrEL) decay and capacitance measurements, further proving the attributions from the deteriorated products in excited states (or highly excited states) and charged‐excited states.

In order to give a more comprehensive discussion on exciplex cohost stability, a series of common adopted electron‐donating carbazole and triphenylamine‐based HTMs, following with electron‐accepting triazine‐based ETMs with adequate thermal stability are involved to give promising exciplex candidates of TCTA:T2T, *N*4,*N*4′‐di(naphthalen‐1‐yl)‐*N*4,*N*4′‐diphenyl‐[1,1′‐biphenyl]‐4,4′‐diamine (NPB):T2T, NPB:10‐(3‐(4,6‐diphenyl‐1,3,5‐triazin‐2‐yl)phenyl)‐10*H*,10'*H*‐spiro[acridine‐9,9'‐anthracen]‐10'‐one (SpiroCO‐mTRZ), NPB:SF2‐TRZ, BCzPh:T2T, and BCzPh:SF2‐TRZ. Their new excited state energies (Figure S1 and Table S1, Supporting Information) distinctly reveal red‐shifted charge transfer emissions of sky‐blue or green colors, confirming the formation of D^+^A^−^ complexes. Interestingly, charge transfer properties were quite different in the involved exciplexes. On the one hand, ^3^CT phosphorescence was not observed in NPB‐based and BCzPh:SF2‐TRZ blended films because triplet local excited states (^3^LE) of NPB (2.26 eV) and SF2‐TRZ (2.46 eV) are much lower than that of exciplex ^3^CT states. Thus, triplets incline decay to ^3^LE via Dexter energy transfer instead. On the other hand, with sufficient ^3^LE to ensure the persistence of ^3^CT and efficient reverse intersystem crossing (RISC) process,^24^ TCTA:T2T and BCzPh:T2T complexes exhibit small Δ*E*_ST_ of 0.04 and 0.02 eV respectively, expecting a RISC‐dominated character from ^3^CT to ^1^CT state.

The totally distinct charge transfer characters motivate us to further investigate their exciton kinetics. As shown in Figure S2 and Table S2 in the Supporting Information, TCTA:T2T and BCzPh:T2T exciplexes show rapid RISC rate constant in ≈10^6^ s^−1^ much greater than the triplet nonradiative rate (*k*_RISC_ ≫ *k*_nr_^T^), confirming an efficient RISC process should dominate the triplet harvesting (**Figure** **1**, Type I). On the contrary, NPB‐based and BCzPh:SF2‐TRZ complexes possess transient decay with only hundreds of nanosecond scale, since low‐lying ^3^LE state is believed to respond for the quick triplet relaxation via Dexter energy transfer (corresponding to *k*_nr_^T^). Therefore, Dexter energy transfer may give greater reaction to passivate reactive triplets than the RISC‐dominated ones (Figure 1, Type II). After doping a red phosphorescent dye bis(2‐phenylquinoline) (2,2,6,6‐tetramethylheptane‐3,5‐dionate)iridium (III) (PQ2Ir), both prompt and delayed decay from exciplex emission dramatically decreased, illustrating a complete Förster resonance energy transfer (FRET, *k*_FRET_ ≈ 10^8^ s^−1^) from ^1^CT state to the phosphorescent guest (Table S2, Supporting Information). An ultrafast FRET makes efforts to eliminate reactive excitons away from the susceptibility of chemical bond dissociation. However, residual cohost triplet excitons still exist in the RISC‐dominated cohosts (Figure S2a and S2e, Supporting Information), suggesting chemical bond would take risks to tolerate possible instability and a higher cohost triplet density could be expected. For giving closed attention to the exciplex cohost itself, a state‐of‐the‐art (τ = ≈1 µs, Figure S2g, Supporting Information) and relatively stable phosphorescent dye PQ2Ir is utilized to avoid severe triplet–triplet annihilation (TTA) from the guest dopant in highly excited states.

**Figure 1:**
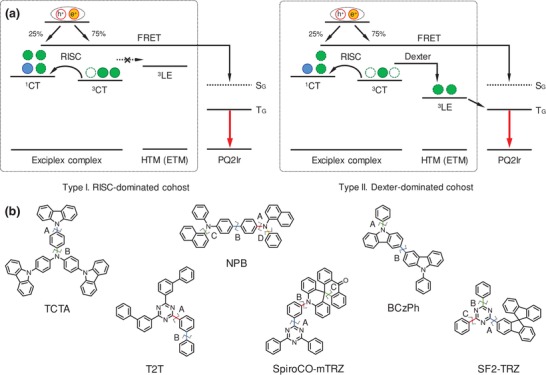
a) Schematic diagrams of proposed energy transfer mechanism in the PQ2Ir:exciplex matrix under electrical excitation (Type I: RISC‐dominated exciplex cohost, Type II: Dexter‐dominated exciplex cohost). b) Chemical structures of the involved exciplex molecules and their studied bond dissociation positions (dash line).

After exploring exciton kinetic properties of the involved exciplex cohosts, the molecular stability analysis is fully investigated then. The involved HTM molecules include C—N and C(sp^2^)—C(sp^2^) bonds in electron‐donating groups (e.g., carbazole, triphenylamine, and acridine derivatives), which are widely adopted. Nevertheless, these *N*‐heterocyclic moieties may expose to a fatal chemical weakness.^25^, ^26^ So, homologous bond dissociation energies (BDEs) were calculated to investigate their molecular stability and possible fragmentation channels. Here, dissociated activation energy (Δ*E*_A_) can be assessed from the Arrhenius equation and the first‐order kinetic equation

(1) $$k_{D}\;=\;k_{0}\;\cdot \;\exp\left(\frac{-\Delta E_{A}}{kT}\right)$$

(2) $$\frac{d\left[\bar{M}\right]}{dt}\;=\;-k_{D}\left[M\right]$$

where *k*_0_ is bond vibration frequency (≈10^12^ s^−1^) and [*M*] represents the molecular density in charged states [*M* ^P^], excited states [M*], charged‐excited states [*M*_P_*], or highly excited states. A commercial LT50 lifetime of 10^6^ h is required and such luminous loss reflects to the defects of nearly 0.1% molecular density. Besides, Joule heat should be considered according to previous studies,^27^, ^28^ and thus the Kelvin temperature parameter *T* in Equation (1) is assumed to be 313 K (40 °C), higher than normal device operation condition to give a more rigid criterion in molecular stability analysis. After that, the corresponding dissociated rate *k*_ds_ = *k*_D_ · [*M*] = 2.78 × 10^8^ s^−1^. Under the degradation in charged states, exciplex cohost molecules are less stable than relevant neutral states. Taking a typical [*M* ^P^] = 5 × 10^19^ cm^−3^, Δ*E*_A_ refers to 1.44 eV. Among the overall BDEs in charged states (Table S4, Supporting Information), chemical stability of anionic state is much weaker than those cationic status, especially for the C—N bond in phenyl‐carbazole moieties, the lowest BDEs can be as low as 1.50 and 1.65 eV in BCzPh and TCTA, respectively. Fortunately, all the consisted exciplex cohost molecules show BDEs still higher than 1.44 eV, indicating only negligible degradation will affect the normal operation when carriers are transporting on their molecular orbital orbits.

Since carriers would generate excitons on ^1^CT and ^3^CT states later (Figure 1), the specific exciton density could motivate excitons from excited state to a higher radical state probably. If this radical energy transfers to a specific inferior bond, bond disruption may take place and detachments will further create quenching defects. According to Equations (1) and (2), the depth of Δ*E*_A_ is susceptible to the related exciton density. Considering the ^1^CT and ^3^CT energies (*S*_E_ and *T*_E_) are much higher than that of the phosphorescent guest, bond dissociated energy difference (Δ_exc_) in excited state is defined as

(3) $$\Delta_{\mathrm{exc},S}\;=\;\mathrm{BDE}\;-\;S_{E}\;-\;\Delta E_{S}$$

(4) $$\Delta_{\mathrm{exc},T}\;=\;\mathrm{BDE}\;-\;T_{E}\;-\;\Delta E_{T}$$

Here, due to the inherent small Δ*E*_ST_, *T*_E_ in Dexter‐dominated exciplexes is assumed to be 0.03 eV lower than *S*_E_. Δ*E*_S_ and Δ*E*_T_ represent dissociated activation energies of ^1^CT and ^3^CT states respectively. When Δ_exc_ < 0, the involved molecules would take risks of suffering bond cleavage and chemical instability.

Under the electrical excitation, singlet density (*N*_S_) and triplet density (*N*_T_) alter as a function of time

(5) $$\frac{dN_{S}}{dt}\;=\;-k_{\mathrm{FRET}}N_{S}\;-\;k_{P}N_{S}\;-\;k_{\mathrm{ISC}}N_{S}\;+\;k_{\mathrm{RISC}}N_{T}\;+\;\frac{J}{4\;\mathrm{de}}$$

(6) $$\frac{dN_{T}}{dt}\;=\;-\;k_{\mathrm{nr}}^{T}N_{T}\;-\;k_{\mathrm{RISC}}N_{T}\;+\;k_{\mathrm{ISC}}N_{S}\;+\;\frac{3\;J}{4\;de}$$

where exciton dynamics rates are adopted according to Tables S2 and S3 in the Supporting Information. Here, assuming the initial electrical excitation energy is equal to optical excitation (i.e., *P* = *J*/d*e* = 7 × 10^17^ cm^−3^, *t* = 0 s),^22^, ^29^ thus receiving Δ*E*_S_ = 1.29 eV and Δ*E*_T_ = 1.32 eV respectively. Since Δ*E*_A_ is a exciton density dependent description, their singlet density threshold *N*_S_ (threshold) and triplet density threshold *N*_T_ (threshold) could be reversely described in the case of Δ*E*_S_ or Δ*E*_T_ ≤ 0 at *t* = 0 s

(7) $$N_{S}\left(\mathrm{threshold}\right)\;=\;\frac{k_{\mathrm{ds}}}{k_{0}\;\cdot \;\exp\left(-\Delta E_{S}/kT\right)}$$

(8) $$N_{T}\left(\mathrm{threshold}\right)\;=\;\frac{k_{\mathrm{ds}}}{k_{0}\;\cdot \;\exp\left(-\Delta E_{T}/kT\right)}$$

Singlet lifetime τ_S_ (threshold) and triplet lifetime τ_T_ (threshold) are finally determined when applying *N*_S_ (threshold) and *N*_T_ (threshold) into Equations (5) and (6), elaborating the time scale for possible weak bond cleavage by ^1^CT and ^3^CT excitons. Molecules would become more susceptible to chemical deterioration and sustain consequent defects when these lifetime thresholds increase.

To analyze the degradation of the involved molecules in excited states, exciton densities as a function of time and bond dissociation energy differences are illustrated in **Figure** **2** and Table S5 in the Supporting Information, respectively. At the moment, excitons are just distributed (*t* = 0 s), ETMs are the most stable stage with very positive Δ_exc_ values. Thus, chemical instability mainly devotes on the detachment of hole‐transporting groups, especially for the acridine group (C–N, position C) in SpiroCO‐mTRZ, an obvious Δ_exc_ weakness (−0.32 eV) is observed. In contrast, the C—N bond from the phenyl‐carbazole group of BCzPh shows an intrinsic strong BDE (3.87 eV), suggesting BCzPh is still impeccable under the influence of reactive ^1^CT and ^3^CT states. Then, when the time moves on (*t* > 0 s), a dramatic decrease for both *N*_S_ and *N*_T_ densities was clearly observed when dispersing PQ2Ir guest into exciplex matrix, confirming FRET indeed assists to release radical energy (Figure 2). More importantly, Dexter‐dominated exciplex cohosts reveal more rapid *N*_S_ and *N*_T_ relaxation within only hundreds of nanosecond than the RISC‐dominated ones, giving advantages to abbreviate τ (threshold) time for hole‐transporting moiety fission and deactivating possible quenching defects. As a result, NPB:T2T and NPB:SF2‐TRZ candidates make efforts to shorten τ_S_ (threshold) and τ_T_ (threshold) than TCTA:T2T, while NPB:SpiroCO‐mTRZ is still maintaining a long threshold in microsecond scale because the acridine group is too weak. Eventually, considering the molecular stability in the overall time scale, a trend of BCzPh:T2T ≈ BCzPh:SF2‐TRZ > NPB:T2T ≈ NPB:SF2‐TRZ > NPB:SpiroCO‐mTRZ ≈ TCTA:T2T can be expected, indicating NPB:SpiroCO‐mTRZ and TCTA:T2T cohosts would face a serious degradation failure.

**Figure 2:**
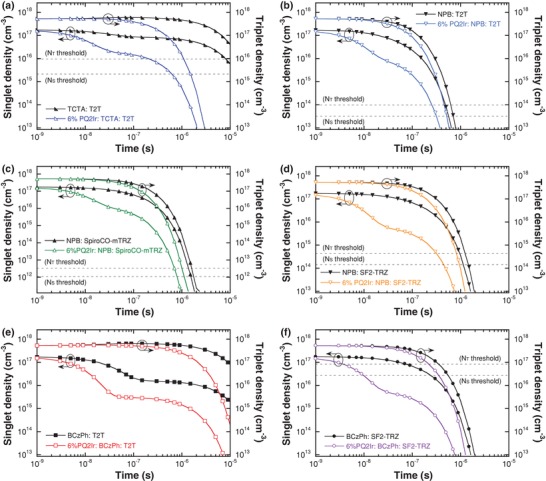
The calculated time‐dependent singlet density and triplet density in a) TCTA:T2T, b) NPB:T2T, c) NPB:SpiroCO‐mTRZ, d) NPB:SF2‐TRZ, e) BCzPh:T2T, and f) BCzPh:SF2‐TRZ exciplex cohosts with the lowest exciton density thresholds (dash line) corresponding to weak bonds.

Somehow, FRET from an exciton to the charged molecule (polaron, D) may induce exciton‐polaron annihilation (EPA) degradation. The charged‐excited molecule (excited polaron, D*) will then dissociate directly (if excited to the purely repulsive term) or via internal conversion and subsequent dissociation from a hot excited stage (“hot‐molecule mechanism”).^30^, ^31^, ^32^, ^33^ According to the excited energies from exciplex or phosphorescent dopant, dissociated activation energy of EPA (Δ*E* ^P^) can be descripted as the difference between charged state BDE (BDE^+/−^) and exciton energy (*E*_exc_)^19^

(9) $$\Delta E^{P}\;=\;{\mathrm{BDE}}^{+/-}\;-\;E_{\mathrm{exc}}$$

As charged‐excited state is extremely short‐lived and difficult to measure clearly, however, their formative probabilities show positive dependence to the polaron and exciton density.^19^ Fortunately, the amount of polarons could be properly controlled by optimizing HTM:ETM ratios.^34^ Thus, we mainly discuss coordinated relationship between average exciton lifetime (<τ>) and Δ*E*_EPA_ here. As shown in Table S6 in the Supporting Information), negatively charged carbazole and triphenylamine fragments again are highly susceptible to the EPA degradation. For instance, extremely low‐lying Δ*E* ^P^ potentials were observed at overall C—N bonds of TCTA molecule even under the PQ2Ir *T*_G_ state. Meanwhile, electron transporting molecules still possess no weakness in anion states. Average exciton lifetime further demonstrates a transient character in Dexter‐dominated cohosts compared with those RISC‐dominated ones. Thus, NPB‐based candidates become more stable with reduced EPA possibilities. In this case, molecular stability of charged‐excited state concludes a tendency of NPB:T2T ≈ NPB:SpiroCO‐mTRZ ≈ NPB:SF2‐TRZ > BCzPh:SF2‐TRZ > BCzPh:T2T > TCTA:T2T eventually, predicting BCzPh:T2T and TCTA:T2T complexes will be more addicted to a charged‐excited failure. Fortunately, the low possibility for a polaron to meet an excited molecule in somewhat reduce the consequence of EPA dissociation than excited state degradation, while TPA dissociation between ^3^CT and polaron is almost vanished due to the spin–orbit forbidden transition.^35^

Finally, in cohost and phosphorescent guest system, guest–guest TTA is a dominant process for highly excited state degradation when host‐guest energy transfer is highly efficient.^36^, ^37^ Hence, degradation in highly excited state is determined using the difference between neutral BDE and twice of phosphorescent triplet energy (*T*_G_), describing as

(10) $$\Delta E_{\mathrm{TTA}}\;=\;\mathrm{BDE}\;-\;2T_{G}$$

Similar to the degradation of molecule in excited state, HTMs still cause distinct negative Δ*E*_TTA_ values and thus promote risks in highly excited state annihilation (Table S7, Supporting Information). Fortunately, short triplet lifetime of PQ2Ir dopant (≈1 µs) gives assistance to solve this crisis, while a broad recombination region with diluent exciton concentrations further make efforts to suppress TTA‐mediated degradation.^16^, ^38^ As TTA rate is still affected by exciton density and neutral BDE of chemical bond, their stability should keep a similar trend as the dissociation in excited state. In conclusion of the overall degradation patterns above, our molecular stability analysis eventually predicted that BCzPh:SF2‐TRZ and NPB:T2T exciplex cohosts would achieve optimized molecular stability with long‐lived operating lifespan (**Figure** **3**), and the most probable dissociation routes may originate from the degradation of hole transporting moieties in charged‐excited state and excited state. In contrast, the most inferior stability should refer to TCTA:T2T cohost due to the critical failure in excited state and charged‐excited state. The remaining complexes of BCzPh:T2T, NPB:SF2‐TRZ, and NPB:SpiroCO‐mTRZ would perform moderate stability with a relation of BCzPh:T2T ≈ NPB:SF2‐TRZ > NPB:SpiroCO‐mTRZ, where NPB:SpiroCO‐mTRZ receive rather severe excited state quenching.

**Figure 3:**
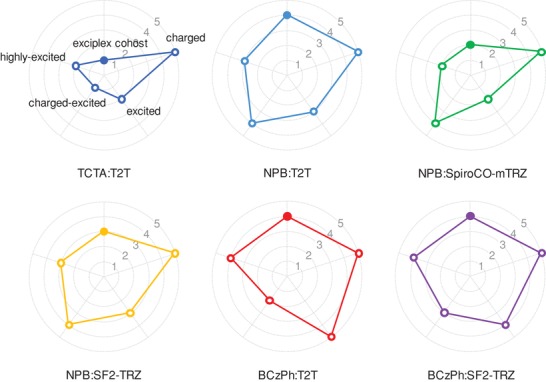
Exciplex cohost stability (solid circle) and degradation of exciplex cohost molecules in charged state, excited state, charged‐excited state, and highly excited state (hollow circle) among the investigated exciplex cohost candidates (stability classification: “1” unstable, “2” quite unstable, “3” fair, “4” quite stable, and “5” very stable).

After molecular stability screening among exciplex cohost candidates and analyzing the dominated dissociation mechanisms, electroluminescence (EL) properties of these complexes were studied to verify our molecular stability prediction with a controlled device configuration: ITO/HATCN (10 nm)/NPB (30 nm)/HTM (10 nm)/6 wt% PQ2Ir:exciplex cohost (25 nm)/T2T (10 nm)/NBPhen (40 nm)/LiF (1 nm)/Al (100 nm), where dipyrazino[2,3‐f:2',3'‐h]quinoxaline‐2,3,6,7,10,11‐hexacarbonitrile (HATCN), NPB, and 2,9‐di (naphthalen‐2‐yl)‐4,7‐diphenyl‐1,10‐phenanthroline (NBPhen) give function as hole injecting layer (HIL), hole transporting layer (HTL), and electron transporting layer (ETL) respectively (**Figure** **4**c). After tuning the HTM:ETM ratio of exciplex cohosts (Table S8, Supporting Information) to achieve optimized performance, the involved OLEDs successfully achieved both high efficiencies and excellent luminous roll‐off control (Figure 4a,b). Their highest EQEs are only determined from the lowest triplet energies of the HTM and ETM components, where the devices based on TCTA:T2T, BCzPh:T2T, and BCzPh:SF2‐TRZ show better EQE values of 19.6%, 20.6%, and 19.8%, respectively. Besides, the low turn‐on voltage follows a similar trend as their corresponding *S*_E_ energies, and thus, higher power efficiencies are expected (**Table** **1**), as compared with conventional host materials.

**Figure 4:**
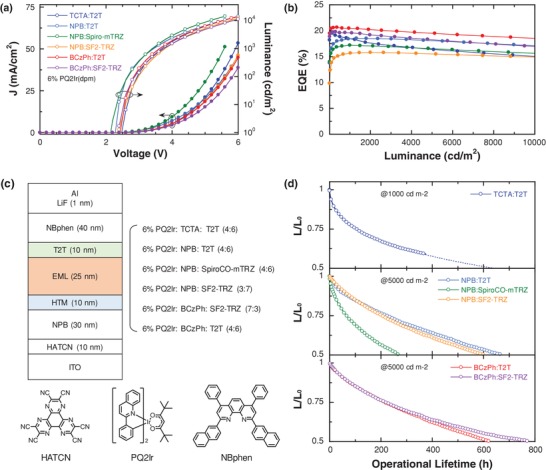
a) Current density–voltage–luminance and b) external quantum efficiency versus luminance characteristics of the exciplex cohost phosphorescent OLEDs. c) Device structures of the involved exciplex cohost candidates and the chemical structures of HATCN, PQ2Ir, and NBphen. d) Operational lifetime of the red phosphorescent OLEDs (the initial luminance (*L*_0_) is 5000 cd m^−2^ except *L*_0_ = 1000 cd m^−2^ for TCTA:T2T device).

**Table 1:** Electroluminescence properties and operational lifetimes of the exciplex cohost phosphorescent OLEDs

Exciplex cohost	*V*_ON_ a) [V]	Voltage/CE/EQE [V cd^−1^ A^−1^ %^−1^]		CIE (*x*, *y*)	LT50b) [h]	Cal. LT50c) [h]
		Maximum	at 1000 cd m^−2^			
TCTA:T2T	2.4	35.5/19.6	3.5/34.0/18.6	(0.60, 0.39)	–	644
NPB:T2T	2.2	33.5/18.5	3.4/33.2/18.4	(0.61, 0.39)	689	9051
NPB:SpiroCO‐mTRZ	2.1	31.1/17.2	3.4/31.0/17.2	(0.61, 0.39)	274	3600
NPB:SF2‐TRZ	2.4	28.0/15.8	3.8/27.7/15.6	(0.61, 0.39)	607	7975
BCzPh:T2T	2.4	36.0/20.6	3.6/35.7/20.5	(0.61, 0.38)	638	8381
BCzPh:SF2‐TRZ	2.3	33.6/19.8	3.7/33.2/19.6	(0.62, 0.38)	774	10 169

^a)^ *V*_ON_ is obtained at 1 cd m^−2^

^b)^ Initial luminance at 5000 cd m^−2^

^c)^ Initial luminance at 1000 cd m^−2^.

More importantly, the operational stability of exciplex cohosts was evaluated and released the same behavior as our previous predication (Figure 4d). Since the HTM:ETM mixing ratio plays significant impacts on charge carrier balance and device lifetime,^34^, ^39^, ^40^ in this case, the HTM:ETM ratios for each exciplex cohost system were carefully optimized while keeping the same controlled device configuration (Table S9, Supporting Information). As predicted previously, the devices based on BCzPh:SF2‐TRZ, and NPB:T2T exhibited the most long‐term working lifetime of 774 and 689 h at an initial luminance of 5000 cd m^−2^. Using a formulation to further estimate LT50 lifetime at a practical luminance of 1000 cd m^−2^

(11) $${\mathrm{LT50}}_{\left(1000\;\mathrm{cd}\;m^{-2}\right)}\;=\;{\left(\frac{{\mathrm{LT50}}_{\left(\mathrm{5000}\;\mathrm{cd}\;m^{-2}\right)}}{{\mathrm{LT50}}_{\left(\mathrm{1000}\;\mathrm{cd}\;m^{-2}\right)}}\right)}^{1.6}$$

LT50 lifetimes of 10 169 and 9051 h could be finally achieved in BCzPh:SF2‐TRZ and NPB:T2T devices respectively, over 14 times longer than that of 644 h in the TCTA:T2T system, which is comparable to the best results in previous academic studies.^38^, ^41^, ^42^, ^43^, ^44^ The inspiring lifetime performance reveals the same tendency as the molecular stability analysis in both RISC‐dominated and Dexter‐dominated exciplex cohosts, successfully achieving operational lifetime predictions without time consuming and complicated device fabrication. This new technique also demonstrates a comprehensive value in screening potentially long‐lived exciplex cohost candidates and therefore shows highly prospect in commercial manufacture.

Dissociated products would further react with neighboring molecules to form defects, such as carrier traps, nonradiative recombination centers and luminescence quenchers.^19^, ^20^, ^45^ Thus, it is of interest to understand their inherent degrading behaviors to further confirm the dominant degradation mechanism by comparing the corresponding pristine and degraded devices. **Figure** **5** illustrates the TrEL decay profiles by inputting a voltage pulse in PQ2Ir emission. The longer TrEL decay tail in aged BCzPh:T2T device is mainly affected by the extended TrEL decay from exciplex cohost (shown in Figure S4, Supporting Information), where most excitons are initially distributed (for other exciplex cohosts, their TrEL decays are shorter than PQ2Ir guests, the influence can be neglected). The aged devices of NPB‐based and TCTA:T2T cohosts decayed clearly compared with the pristine ones, clearly indicating the formation of exciton quenchers during persistent electrical operation. On the contrary, only small distinction was found in BCzPh‐based OLEDs. By further measuring the number of luminous defects, areas of the TrEL profiles and their relative quenching rates (*v*_Q_) were calculated over the time period to quantitatively discuss the quenching characteristics. As the result shown in Table S10 in the Supporting Information), the BCzPh:T2T and BCzPh:SF2‐TRZ candidates possess much slower quenching rates (3.86 × 10^−4^ and 6.27 × 10^−4^%·h^−1^) than that of NPB:SpiroCO‐mTRZ and TCTA:T2T cohosts, suggesting exciton quenchers may strongly correlate with the dissociation consequence of mole‐cules in excited and highly excited states. Generally, these dissociated products are taken place within the exciton generation region and possible radical cleavage fragments (carbazole, acridine, etc.) would connect with the adjacent molecules to generate long π‐conjugation quenching products.^25^, ^26^, ^46^ Hence, homolytic cleavage reactions within NPB, TCTA, and SpiroCO‐mTRZ HTMs should take response for the accumulation of exciton quenchers and luminous degradation consequence.

**Figure 5:**
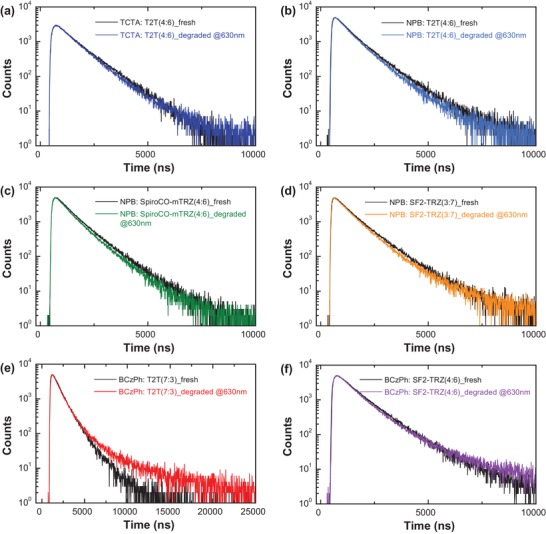
Transient electroluminescence profiles of the pristine and degraded OLEDs with a) TCTA:T2T, b) NPB:T2T, c) NPB:SpiroCO‐mTRZ, d) NPB:SF2‐TRZ, e) BCzPh:T2T, and f) BCzPh:SF2‐TRZ exciplex cohosts at the wavelength of PQ2Ir emission.

The corruptions of hole‐transporting moiety may also induce carrier injection and transport changes. Current density–voltage (*J*–*V*) characteristics of the pristine and degraded OLEDs were fully investigated (**Figure** **6**), and the carrier injection curves shifted toward to higher voltage region, showing a probably interfacial aging and parasitic injection barrier appear to result in the voltage increase. To figure out whether this phenomenon comes from a consequence of the HTM or ETM components, capacitance–frequency (*C*–*F*) measurement was carried out (Figure S5, Supporting Information). Here, relaxation frequency (*f*_r_) is defined as^47^

(12) $$f_{r}\;=\;\frac{1}{2\pi }\;\cdot \;\frac{1}{R_{\mathrm{HTM}}\;\cdot \;\left(C_{\mathrm{HTM}}\;+\;C_{\mathrm{ETM}}\right)}$$

where *R*_HTM_, *C*_HTM_, and *C*_ETM_ are the resistance of HTM, and capacitances of HTM and ETM, respectively. Relaxation frequency for the aged devices is much smaller than their corresponding fresh ones with *C*_ETM_ values dramatically decreased simultaneously, suggesting HTMs would take response for the interfacial and carrier injection inferior. Possible internal mechanism is that dissociative carbazole or triphenylamine fragments from HTM molecules may primarily create hole traps near the HTM/EML interface, and then carry the fixed positive charge after hole trapping. The charge accumulation and change in electric field profile eventually lead to a gradual increase of the driving voltage and parasitic hole injection barrier.^25^ To our surprise, the BCzPh‐based exciplexes are more impressible to hole degradation, which is electrochemical stable within excited and highly excited states. Therefore, it is reasonable to conclude that this hole trap formation would be highly related to the EPA aggregation. Capacitance–voltage (*C*–*V*) measurement further indicates the hole injection changes, generally, the first capacitance peak nearby built‐in voltage (*V*_bi_) belongs to carrier injection, as summarized in Figure 6 and Table S11 in the Supporting Information. Those exciplex cohosts with severe EPA annihilation suffer more injection decay and larger carrier trap generation rate (*v*_CT_), while the NPB‐based cohosts are benefitted from a rather quick exciton relaxation and stabilized BDEs in charged states. In addition, since device stabilities highly depend on HTM:ETM ratios, *v*_CT_ properties with various BCzPh:SF2‐TRZ ratios were also calculated. BCzPh:SF2‐TRZ in a ratio of 4:6 showed longer device stability and passivated *v*_CT_ rate compared with the other proportions (Table S11, Supporting Information), indicating a proper control of HTM:ETM ratios indeed takes good management of restraining the polaron‐induced EPA quenching and extending operational stabilities.

**Figure 6:**
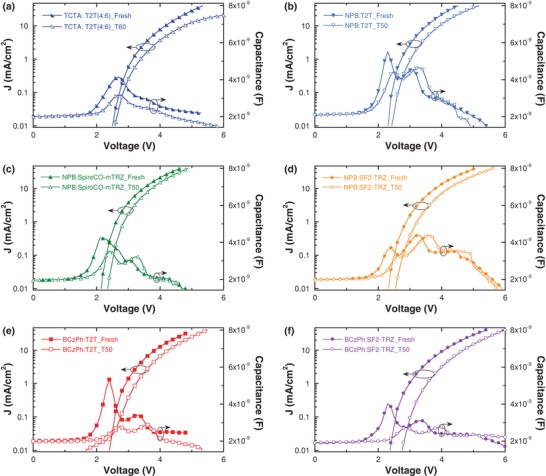
Current density–voltage and capacitance–voltage (applied frequency *f*_0_ of 1 kHz) characteristics of the pristine and degraded OLEDs with a) TCTA:T2T, b) NPB:T2T, c) NPB:SpiroCO‐mTRZ, d) NPB:SF2‐TRZ, e) BCzPh:T2T, and f) BCzPh:SF2‐TRZ exciplex cohosts.

In summary, a feasible procedure was demonstrated to give precise molecular stability prediction for the exciplex cohosts and analyze dominated degradation mechanism by coordinating dissociated activation energy description with exciton density as a function of time. As the result, molecular degradation in excited states (or highly excited states) and charged‐excited states shows significant contributions to induce inherent chemical instability and bond cleavage of the hole transporting moieties. This crisis could be properly solved by means of adopting invincible chemical stability and rapid Dexter‐dominated exciton relaxation managements. The following red phosphorescent OLEDs reveal a well agreement with the simulated molecular stability analysis, achieving a high maximum EQE of 19.8% and LT50 of 10 169 h in the predicted long‐term BCzPh:SF2‐TRZ cohosts, which is ≈16 times longer than the reference group TCTA:T2T. Transient electroluminescence decays further confirm that excited‐state instability is an initial source for luminescence quenchers, while hole traps accumulation may be attributed to the deteriorated products of charged‐excited state that cause a hole injection failure eventually. Although the device lifetimes reported here are still behind the criteria for commercial applications, further improvement could be achieved by developing new cohost molecules with more eminent electrochemical stability. More importantly, this new achievement gives a feasible guidance to screen the potentially long lifetime exciplex candidates with decreased processing time and expenses.

## Experimental Section

*Quantum Chemical Calculations*: The program Gaussian 09 was used for all the calculations described in this paper. The single point energy calculations of each fragment (including radical and charged species) were performed using DFT with the B3LYP/6‐31G (d) level. The fragments were picked from the entire molecules and without further optimization. The unrestricted formalism was used for the geometry optimization and frequency calculations of neutral molecules, cation radicals, and anion radicals. The BDE was calculated according to the enthalpy change in the corresponding reaction of homolytic cleavage of a single bond in the gas phase at 298 K and 1 atm. Calculations on the ground state configurations of the neutral and charged molecules were carried out using TDDFT theory at the level of B3LYP/6‐31G (d). Frequency analysis was performed to ensure that local minimum was found.

*General*: UV–vis absorption spectra were recorded on a HP 8453 spectrophotometer. Photoluminescence (PL) spectra were measured using a Jobin‐Yvon spectrofluorometer. Organic films for optical measurements were evaporated onto precleaned quartz substrates via thermal evaporation under high vacuum of <1 × 10^−4^ Pa. Photoluminescence quantum yields (PLQYs) of solid films were recorded at room temperature by using a photoluminescence quantum yield measurement system Hamamatsu Photonics C9920‐02. Transient photoluminescence characteristics of the evaporation deposited films were collected by Hamamatsu Photonics C4334 at 300 K. Transient electroluminescence characteristics of encapsulated devices were detected by Edinburgh FL980 with a 10 V signal pulse (pulse width = 250 ns, frequency = 10 kHz). The capacitance–voltage and capacitance–frequency measurements were performed using HP 4192A LF impedance analyzer with 0.05 V perturbation oscillation signal.

*Materials*: The presented transporting materials NPB and T2T were purchased from Xi'an Polymer Light Technology Co. Ltd. (Xi'an, Shaanxi province, China), while HATCN, TCTA, and NBPhen were used as received from Luminescence Technology Corp. SF2‐TRZ was synthesized according to the reported procedures.^48^ SpiroCO‐mTRZ was synthesized in the laboratory and will be reported elsewhere.

*Device Preparation and Measurements*: Glass substrates precoated with a 95 nm thin layer of indium tin oxide (ITO) with a sheet resistance of 15–20 Ω sq^−1^ were thoroughly cleaned by ultrasonic of various detergents and treated with O_2_ plasma. OLED devices were fabricated onto the cleaned ITO‐coated glass substrates by thermal evaporation under high vacuum (<2 × 10^−4^ Pa). Deposition rates were 1 Å s^−1^ for organic materials, 0.1 Å s^−1^ for LiF layer, and 7–8 Å s^−1^ for Al film, respectively. After fabrication, the devices were immediately encapsulated with a glass cover using epoxy glue in nitrogen‐filled glove boxes (O_2_, <1 ppm, H_2_O, <1 ppm). A piece of commercial calcium oxide desiccant (Dynic Co., Japan) was placed in the encapsulated package. Then, OLED devices at once were exposed to ultraviolet ray with a shadow mask to protect the emitting area. The current density–luminance–voltage (*J*–*V*–*L*) characteristics were measured by Keithley 2400 and Konica Minolta CS‐200 electroluminescence measurement system. EL spectra of the devices were recorded by Ocean Optics spectrometer USB2000+ and power supply Keithley 2400. Device operational stability was measured using a multichannel device lifetime system to obtain the electroluminescence property of device stability at a constant current density mode and maintain at room temperature of 23 ± 1 °C.

## Conflict of Interest

The authors declare no conflict of interest.

## Supporting information

Supplementary
