# Supplementary material for: Predicting Operational Stability for Organic Light‐Emitting Diodes with Exciplex Cohosts
Source: Adv Sci (Weinh). 2019 Feb 13;6(7):1802246. doi: 10.1002/advs.201802246 (PMC6446740; doi:10.1002/advs.201802246)
Supplement: Supplementary file 1 — Supplementary [file ADVS-6-1802246-s001.pdf]

Copyright WILEY-VCH Verlag GmbH & Co. KGaA, 69469 Weinheim, Germany, 2019.

Supporting Information

for *Adv. Sci.,* DOI: 10.1002/advs.201802246

Predicting Operational Stability for Organic Light-Emitting Diodes with Exciplex Cohosts

*Zhiheng Wang, Mengke Li, Lin Gan, Xinyi Cai, Binbin Li, Dongcheng Chen, and Shi-Jian Su**

Copyright WILEY-VCH Verlag GmbH & Co. KGaA, 69469 Weinheim, Germany, 2018.

Supporting Information

Predicting operational stability for organic light-emitting diodes with exciplex cohosts

Zhiheng Wang, Mengke Li, Lin Gan, Xinyi Cai, Binbin Li, Dongcheng Chen, Shi-Jian Su*

State Key Laboratory of Luminescent Materials and Devices and Institute of Polymer Optoelectronic Materials and Devices, South China University of Technology, Guangzhou, 510640, P. R. China
E-mail: mssjsu@scut.edu.cn

Table of Contents

Method S1. Exciton dynamic parameters. 3

Figure S1. UV-vis absorption and PL spectra of the exciplexes. 4

Figure S2. Transient photoluminescence dedays of the codeposited films. 5

Figure S3. Electroluminescence spectra of the exciplex cohost phosphorescent OLEDs. 6

Figure S4. Transient electroluminescence profiles of the pristine and degraded OLEDs at exciplex host emission. 7

Figure S5. Capacitance-frequency characteristics in the pristine and degraded OLEDs. 8

Table S1. UV-vis absorption and excited state energies of the exciplexes and their components. 9

Table S2. Exciton dynamic properties and quantum efficiency of the exciplexes. 9

Table S3. Exciton dynamic properties of the PQ2Ir: exciplex blended films. 10

Table S4. Average bond dissociation energies (BDEs) of the involved molecules. 10

Table S5. Calculated bond dissociated energy differences (Δ_{exc}), exciton density thresholds N(threshold) and exciton lifetime thresholds τ(threshold). 11

Table S6. Calculated bond dissociated energies (ΔE^{P}) and average exciton lifetimes $<\tau>$ 12

Table S7. Calculated bond dissociated energies (ΔE_{TTA}) of the involved molecules. 14

Table S8. Electroluminescence properties of the exciplex cohost phosphorescent OLEDs in various HTM: ETM ratios. 15

Table S9. Operational stability of exciplex cohost OLEDs with various HTM: ETM ratios. 16

Table S10. TrEL decay changes and relative exciton quenching rates (v_Q) in the pristine and degraded OLEDs. 17

Table S11. Carrier injection changes and the corresponding carrier trap generation rate in the pristine and degraded exciplex cohost devices. 17

Method S1. Exciton dynamic parameters. Reverse intersystem crossing rate constant (k_{RISC}) can be determined as follow:

$$k_{RISC} = \frac{k_d k_p}{k_{ISC}} \frac{\phi_d}{\phi_p}, \quad \text{(S1)}$$

Where k_p and k_d are prompt and delayed fluorescence rate constant, ϕ_p and ϕ_d are prompt and delayed fluorescence efficiency, which are estimated from the total photoluminescence quantum yields (PLQYs). The intersystem crossing rate constant (k_{ISC}) and nonradiative rate constant are calculated by the following relation

$$k_{ISC} = (1 - \phi_p) k_p, \quad \text{(S2)}$$

$$k_{nr}^T = k_d - \phi_p k_{RISC}, \quad \text{(S3)}$$

$$k_p = \frac{\phi_p}{\tau_p}, \quad \text{(S4)}$$

$$k_d = \frac{\phi_d}{\tau_d}. \quad \text{(S5)}$$

In equations (4) and (5), τ_p and τ_d are prompt and delayed lifetimes in HTM: ETM mixed films, which can be fitted from the time-resolved PL decay curves. The k_{FRET} in the 6 wt.% PQ2Ir: HTM: ETM blended films is evaluated from the prompt part of the decay curves using the equations below,

$$k_d = \frac{1}{\tau_p'} - \frac{1}{\tau_p}, \quad \text{(S6)}$$

where τ'_p is the prompt lifetime in 6 wt.% PQ2Ir: HTM: ETM blended films.

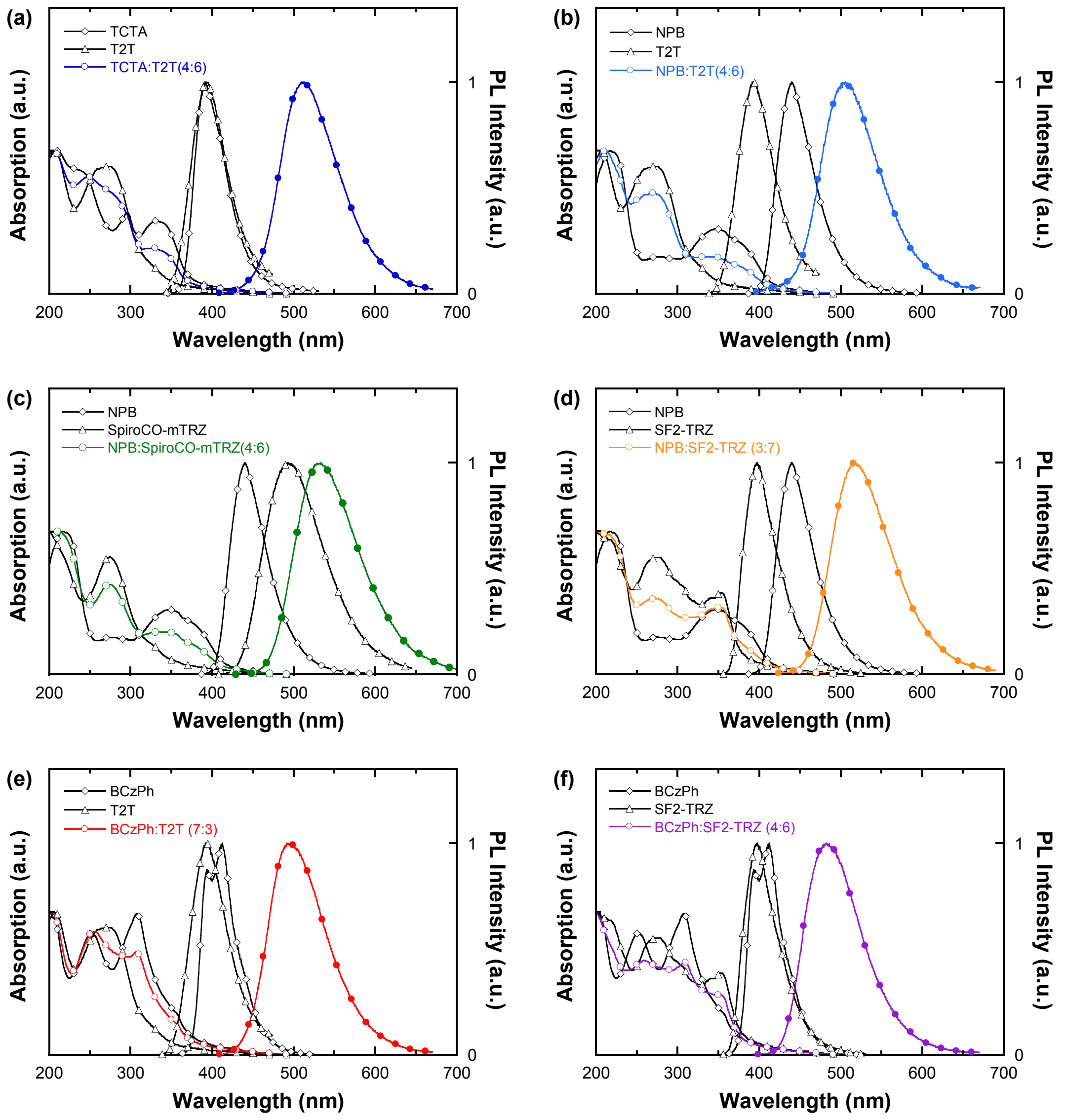

Figure S1. UV-vis absorption and PL spectra of the binary blended films of a) TCTA: T2T, b) NPB: T2T, c) NPB: SpiroCO-mTRZ, d) NPB: SF2-TRZ, e) BCzPh: T2T and f) BCzPh: SF2-TRZ and their corresponding components.

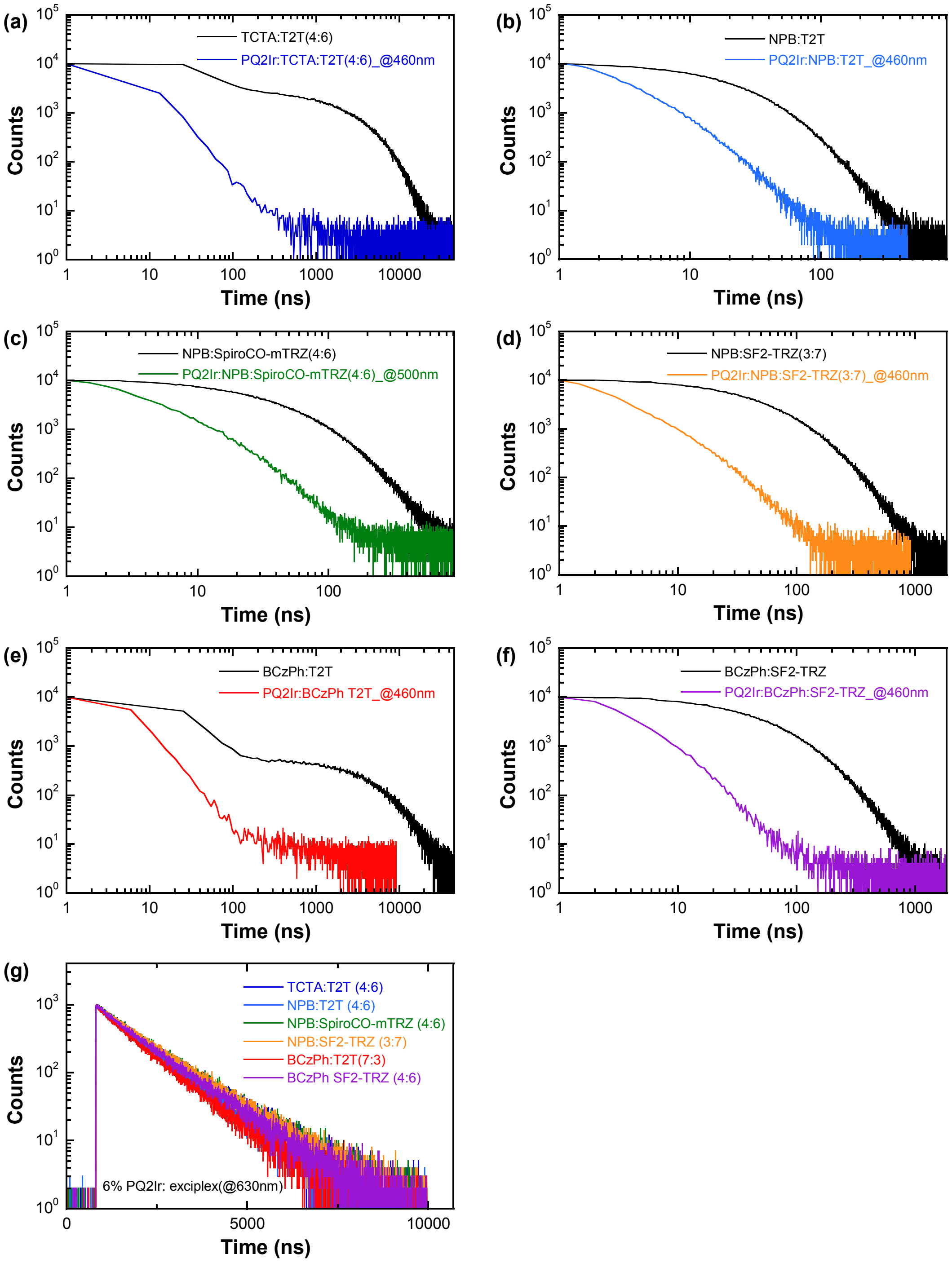

Figure S2. Transient photoluminescence decays of the a) TCTA: T2T, b) NPB: T2T, c) NPB: SpiroCO-mTRZ, d) NPB: SF2-TRZ, e) BCzPh: T2T and f) BCzPh: SF2-TRZ exciplexes, and their corresponding 6 wt.% PQ2Ir: exciplex codeposited films from exciplex cohosts emission and g) PQ2Ir emission.

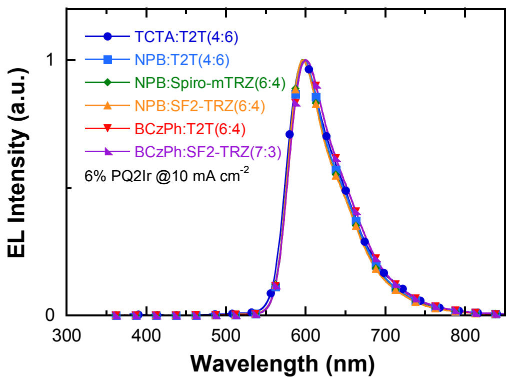

Figure S3. Electroluminescence (EL) spectra of the exciplex cohost phosphorescent OLEDs measured at a current density of 10 mA cm^{-2}.

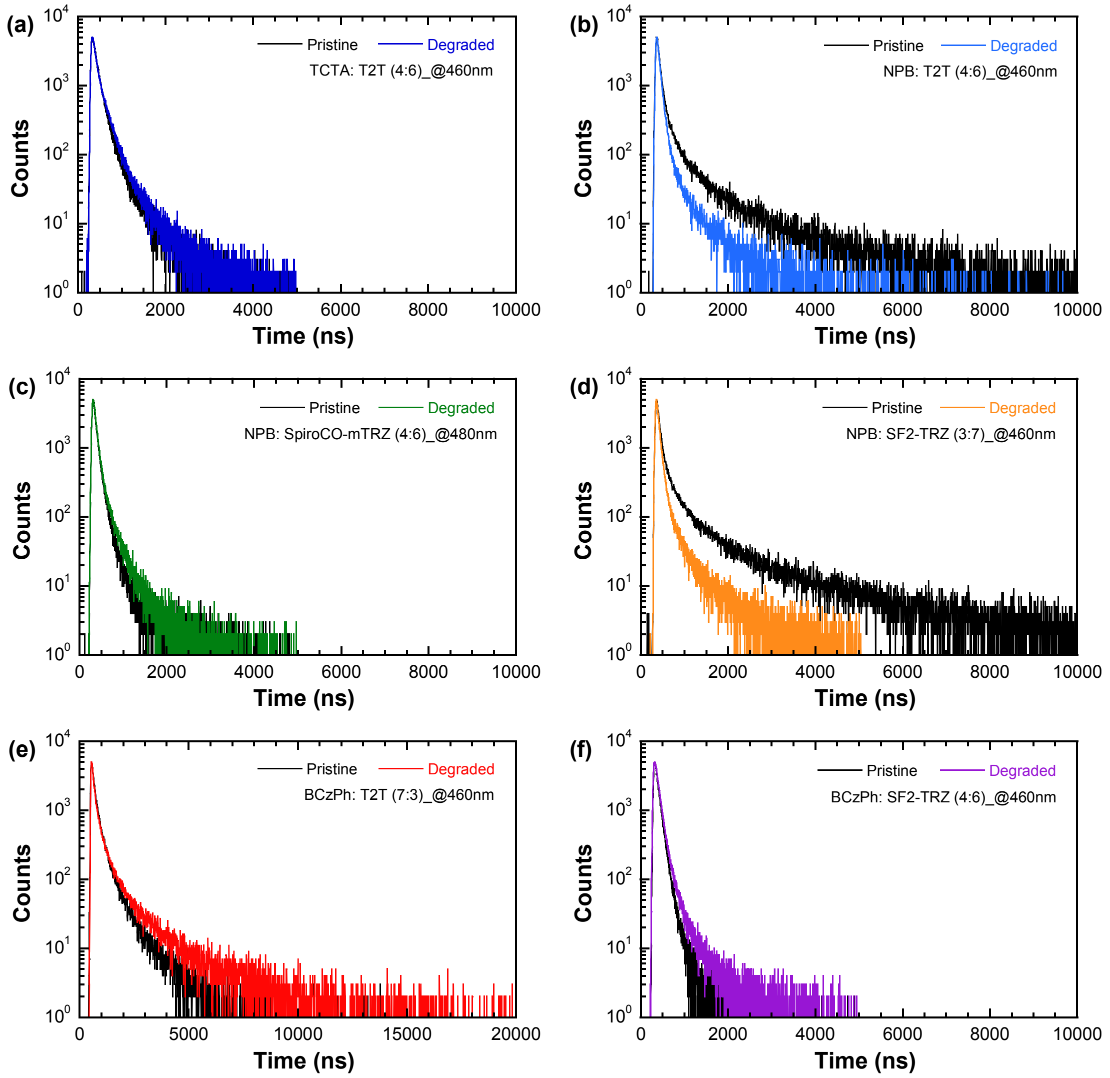

Figure S4. Transient electroluminescence profiles of the pristine and degraded phosphorescent OLEDs with a) TCTA: T2T, b) NPB: T2T, c) NPB: SpiroCO-mTRZ, d) NPB: SF2-TRZ, e) BCzPh: T2T and f) BCzPh: SF2-TRZ exciplex cohosts at the wavelength of exciplex emission.

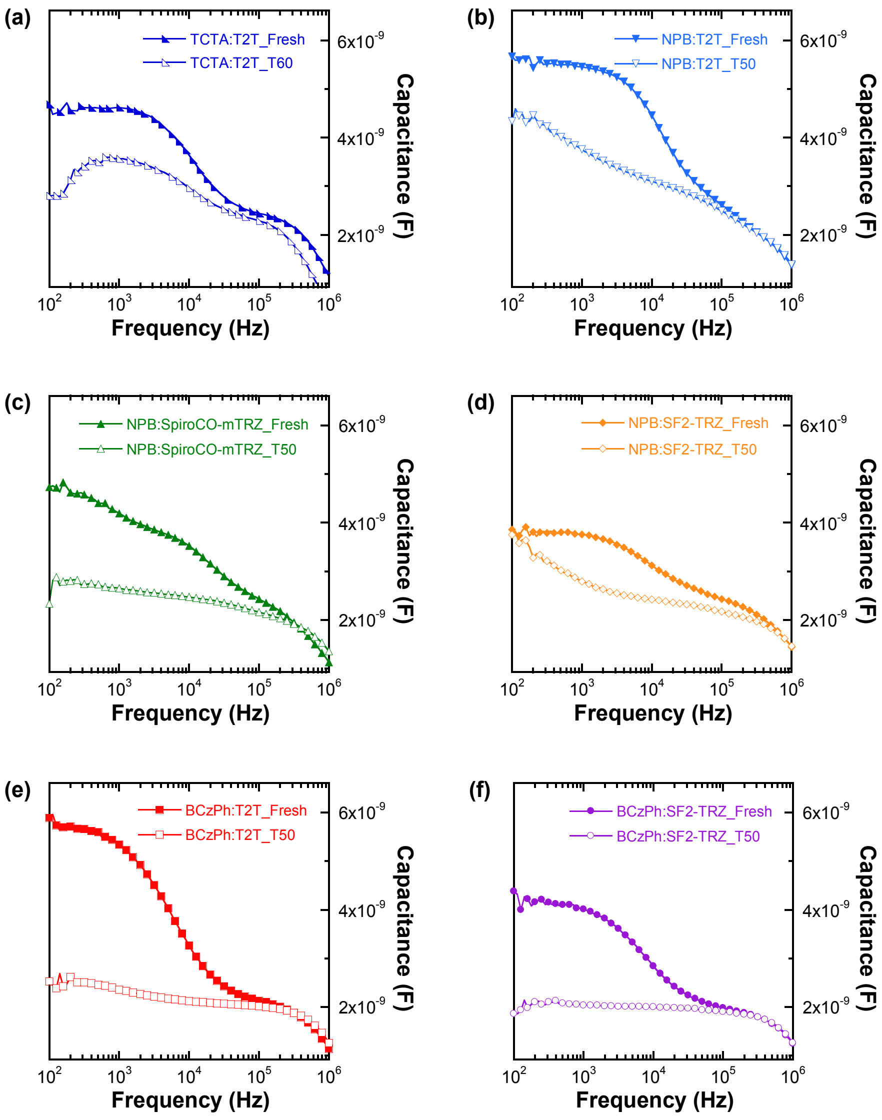

Figure S5. Capacitance-frequency characteristics of the pristine and degraded a) TCTA: T2T, b) NPB: T2T, c) NPB: SpiroCO-mTRZ, d) NPB: SF2-TRZ, e) BCzPh: T2T and f) BCzPh: SF2-TRZ OLEDs at applied voltages (V_0) of 2.7, 2.3, 2.1, 2.3, 2.4 and 2.3 V.

Table S1. UV-vis absorption and excited state energies of the binary blended exciplexes and the corresponding components in solid films.

Compound	$\lambda_{max, abs}$ (nm)	$\lambda_{max, PL}$ (nm)	S_1/ T_1 (eV) [a]	ΔE_{ST} (eV)
NPB	350, 279	440	2.84/ 2.25	0.59
TCTA	335, 295	392	3.18/ 2.72	0.46
T2T	327, 273	394	3.10/ 2.62	0.48
SpiroCO-mTRZ	373, 330, 275	494	2.54/ 2.52	0.02
BCzPh	306, 247	393, 412	3.16/ 2.59	0.57
SF2-TRZ	355, 301, 272	397	3.21/ 2.46	0.75
TCTA: T2T	330, 277	511	2.50/ 2.46	0.04
NPB: T2T	348, 270	507	2.45/ -	-
NPB: SpiroCO-mTRZ	350, 275	532	2.37/ -	-
NPB: SF2-TRZ	351, 273	517	2.41/ -	-
BCzPh: T2T	355, 308, 256	496	2.54/ 2.52	0.02
BCzPh: SF2-TRZ	355, 306, 260	482	2.63/ -	-

[a] "-" means T_1 is not observed from 77K phosphorescent spectrum.

Table S2. Exciton dynamic properties and photoluminescence quantum yields of the investigated exciplexes.

Exciplex	PLQY (%)	χ^2	τ_p/ A1 [a] (ns/ %)	τ_d/ A2 [a] (μs/ %)	k_p ($\times10^5$ s^{-1})	k_{ISC} ($\times10^5$ s^{-1})	k_{RISC} ($\times10^5$ s^{-1})	k_{nr}^T ($\times10^5$ s^{-1})
TCTA: T2T	46.9	1.02	30.2/ 6.4	2.92/ 93.6	9.9	322	47	1.7
NPB: T2T	4.2	0.88	22.9/ 80.1	0.06/ 19.9	15	423	41	157
NPB: SpiroCO-mTRZ	5.4	1.13	40.1/ 71.8	0.11/ 28.2	9.7	240	37	90
NPB: SF2-TRZ	8.8	0.95	52.0/ 77.7	0.15/ 22.3	13	180	24	65
BCzPh: T2T	37.6	1.06	29.1/ 22.0	4.67/ 78.0	28	315	8.3	1.5
BCzPh: SF2-TRZ	12.6	0.92	52.5/ 75.9	0.16/ 24.1	18	172	23	63

[a] A1 and A2 are ratio of prompt and delayed part respectively.

Table S3. Exciton dynamic properties of the PQ2Ir: exciplex blended films.

Exciplex host	χ^2	τ'_p/ A1 (ns/ %)	τ'_d/ A2 (μs/ %)	τ'^{-1}_p ($\times10^7$ s^{-1})	k_{FRET} ($\times10^7$ s^{-1})
6 wt.% PQ2Ir: TCTA: T2T (4: 6)	0.81	9.06/ 95.2	0.711/ 4.8	11.0	7.72
6 wt.% PQ2Ir: NPB: T2T (4: 6)	0.83	5.02/ 74.2	0.018/ 25.8	19.9	15.5
6 wt.% PQ2Ir: NPB: SpiroCO-mTRZ (4: 6)	1.21	6.78/ 84.7	0.035/ 15.3	14.7	12.2
6 wt.% PQ2Ir: NPB: SF2-TRZ (3: 7)	0.82	5.09/ 86.0	0.031/ 14.0	19.6	17.7
6 wt.% PQ2Ir: BCzPh: T2T (7: 3)	1.18	6.88/ 92.1	0.633/ 7.9	14.5	11.1
6 wt.% PQ2Ir: BCzPh: SF2-TRZ (4: 6)	0.77	4.73/ 97.0	0.059/ 3.0	21.1	19.2

Table S4. Average bond dissociation energies (BDEs) of the involved molecules in neutral, cationic and anionic states.

material	Bond (Position)	state	bond dissociated reaction	average BDE (eV)
TCTA	C-N (A)	neutral	TCTA→ [TCTA- Cz]· + Cz·	3.95
		cationic	$TCTA^+$→ $[TCTA- Cz]^+$ + Cz·	5.24
		anionic	$TCTA^-$→ [TCTA- Cz] ·+ Cz-	2.04
	C-N (B)	neutral	TCTA→ [TCTA- PhCz]· + PhCz·	3.67
		cationic	$TCTA^+$→ $[TCTA- PhCz]^+$ + PhCz·	4.15
		anionic	$TCTA^-$→ $[TCTA- PhCz]^-$ + PhCz·	1.65
NPB	C-N (A)	neutral	NPB→ [NPB- NPA]·+ NPA·	3.75
		cationic	NPB^+→ [NPB- NPA]· + NPA^+	5.23
		anionic	NPB^-→ [NPB- NPA]· + NPA^-	2.47
	C-C (B)	neutral	NPB→ [NPB- PhNPA]· + PhNPA·	5.33
		cationic	NPB^+→ [NPB- PhNPA]·+ $PhNPA^+$	7.28
		anionic	NPB^-→ [NPB- PhNPA]·+ $PhNPA^-$	4.89
	C-N (C)	neutral	NPB→ [NPB- Na]·+ Na·	3.51
		cationic	NPB^+→ $[NPB- Na]^+$ + Na·	3.95
		anionic	NPB^-→ $[NPB- Na]^-$ + Na·	2.13
	C-C (D)	neutral	NPB→ [NPB- Ph]· + Ph·	3.66
		cationic	NPB^+→ $[NPB- Ph]^+$ + Ph·	4.11
		anionic	NPB^-→ $[NPB- Ph]^-$+ Ph·	2.18
BCzPh	C-N (A)	neutral	BCzPh→ [BCzPh- Ph]· + Ph·	3.87
		cationic	$BCzPh^+$→ $[BCzPh- Ph]^+$ + Ph·	4.24
		anionic	$BCzPh^-$→ $[BCzPh- Ph]^-$ + Ph·	1.50
	C-C (B)	neutral	BCzPh→ [BCzPh- PhCz]· + PhCz·	4.79
		cationic	$BCzPh^+$→ [BCzPh- PhCz]· + $PhCz^+$	6.84

		anionic	BCzPh$^-$→ [BCzPh- PhCz]· + PhCz$^-$	3.89
T2T	C-C (A)	neutral	T2T→ [T2T- BPh]· + BPh·	5.19
		cationic	T2T$^+$→ [T2T- BPh]$^+$ + BPh·	6.03
		anionic	T2T$^-$→ [T2T- BPh]$^-$ + BPh·	5.09
	C-C (B)	neutral	T2T→ [T2T- Ph]· + Ph·	5.20
		cationic	T2T$^+$→ [T2T- Ph]$^+$ + Ph·	6.25
		anionic	T2T$^-$→ [T2T- Ph]· + Ph$^-$	5.71
SpiroCO-mTRZ	C-C (A)	neutral	SpiroCO-mTRZ→ [SpiroCO-mTRZ- PhSpiroCO]· + PhSpiroCO·	5.19
		cationic	SpiroCO-mTRZ$^+$→ [SpiroCO-mTRZ- PhSpiroCO]· + PhSpiroCO$^+$	6.64
		anionic	SpiroCO-mTRZ$^-$→ [SpiroCO-mTRZ- PhSpiroCO]$^-$ + PhSpiroCO·	5.58
	C-N (B)	neutral	SpiroCO-mTRZ→ [SpiroCO-mTRZ- SpiroCO] ·+ SpiroCO·	3.34
		cationic	SpiroCO-mTRZ$^+$→ [SpiroCO-mTRZ- SpiroCO]· + SpiroCO$^+$	3.71
		anionic	SpiroCO-mTRZ$^-$→ [SpiroCO-mTRZ- SpiroCO]· + SpiroCO$^-$	2.64
	C-C (C)	neutral	SpiroCO-mTRZ→ [SpiroCO-mTRZ- BP]· + BP·	3.50
		cationic	SpiroCO-mTRZ$^+$→ [SpiroCO-mTRZ- BP]$^+$ + BP·	3.47
		anionic	SpiroCO-mTRZ$^-$→ [SpiroCO-mTRZ- BP]· + BP$^-$	3.36
SF2-TRZ	C-C (A)	neutral	SF2-TRZ→ [SF2-TRZ- SF]· + SF·	5.22
		cationic	SF2-TRZ$^+$→ [SF3-TRZ- SF]· + SF$^+$	6.39
		anionic	SF2-TRZ$^-$→ [SF5-TRZ- SF]· + SF$^-$	5.24
	C-C (B)	neutral	SF2-TRZ→ [SF2-TRZ- 1'-Ph]· + 1'-Ph·	5.18
		cationic	SF2-TRZ$^+$→ [SF2-TRZ- 1'-Ph]$^+$ + 1'-Ph·	5.89
		anionic	SF2-TRZ$^-$→ [SF2-TRZ- 1'-Ph]· + 1'-Ph$^-$	5.74
	C-C (C)	neutral	SF2-TRZ→ [SF2-TRZ- 5'-Ph]· + 5'-Ph·	5.17
		cationic	SF2-TRZ$^+$→ [SF2-TRZ- 5'-Ph]$^+$ + 5'-Ph·	5.92
		anionic	SF2-TRZ$^-$→ [SF2-TRZ- 5'-Ph]$^-$ + 5'-Ph·	5.17

Table S5. Calculated bond dissociated energy differences (Δ_{exc}), exciton density thresholds N(threshold) and exciton lifetime thresholds τ(threshold) of the involved molecules in excited state degradation.

Exciplex	material	bond (position)	$\Delta_{exc,S}$ (eV)	$\Delta_{exc,T}$ (eV)	N_S(threshold) (cm^{-3})	N_T(threshold) (cm^{-3})	τ_S(threshold) (ns)	τ_T(threshold) (ns)
TCTA: T2T	TCTA	C-N (A)	0.16	0.17	6.8×10^{19}	3.0×10^{20}		
		C-N (B)	-0.12	-0.11	2.2×10^{15}	9.5×10^{15}	618	1110
	T2T	C-N (A)	1.40	1.41	5.3×10^{39}	2.3×10^{40}		
		C-C (B)	1.41	1.42	9.5×10^{39}	4.2×10^{40}		
NPB: T2T	NPB	C-N (A)	0.01	0.01	2.2×10^{17}	6.7×10^{17}		
		C-C (B)	1.59	1.59	7.3×10^{42}	2.2×10^{43}		
		C-N (C)	-0.23	-0.23	3.2×10^{13}	9.8×10^{13}	318	460

		C-C (D)	-0.08	-0.08	8.0×10^{15}	2.4×10^{16}	29	167
	T2T	C-C (A)	1.45	1.45	3.4×10^{40}	1.0×10^{41}		
		C-C (B)	1.46	1.46	6.0×10^{40}	1.8×10^{41}		
NPB: SpiroCO-mTRZ	NPB	C-N (A)	0.09	0.09	4.3×10^{18}	1.3×10^{19}		
		C-C (B)	1.67	1.67	1.4×10^{44}	4.3×10^{44}		
		C-N (C)	-0.15	-0.15	6.3×10^{14}	1.9×10^{15}	265	471
		C-C (D)	0.00	0.00	1.5×10^{17}	4.7×10^{17}		
	SpiroCO-mTRZ	C-C (A)	1.53	1.53	6.9×10^{41}	2.1×10^{42}		
		C-N (B)	-0.32	-0.32	1.1×10^{12}	3.3×10^{12}	792	1000
		C-C (C)	-0.17	-0.17	3.7×10^{14}	1.1×10^{15}	308	514
NPB: SF2-TRZ	NPB	C-N (A)	0.05	0.05	9.8×10^{17}	3.0×10^{18}		
		C-C (B)	1.63	1.63	3.2×10^{43}	9.7×10^{43}		
		C-N (C)	-0.19	-0.19	1.4×10^{14}	4.3×10^{14}	448	823
		C-C (D)	-0.04	-0.04	3.5×10^{16}	1.1×10^{17}	9	187
	SF2-TRZ	C-C (A)	1.52	1.52	5.5×10^{41}	1.7×10^{42}		
		C-C (B)	1.48	1.48	1.0×10^{41}	3.1×10^{41}		
		C-C (C)	1.47	1.47	7.3×10^{40}	2.2×10^{41}		
BCzPh: SF2-TRZ	BCzPh	C-N (A)	-0.05	-0.05	2.7×10^{16}	8.3×10^{16}	10	222
		C-C (B)	0.87	0.87	1.9×10^{31}	5.9×10^{31}		
	SF2-TRZ	C-C (A)	1.30	1.30	1.6×10^{38}	4.8×10^{38}		
		C-C (B)	1.26	1.26	2.9×10^{37}	8.9×10^{37}		
		C-C (C)	1.25	1.25	2.1×10^{37}	6.4×10^{37}		
BCzPh: T2T	BCzPh	C-N (A)	0.04	0.03	7.7×10^{17}	1.6×10^{18}		
		C-C (B)	0.96	0.95	5.4×10^{32}	1.1×10^{33}		
	T2T	C-N (A)	1.36	1.35	1.2×10^{39}	2.5×10^{39}		
		C-C (B)	1.37	1.36	2.1×10^{39}	4.5×10^{39}		

Table S6. Calculated bond dissociated energies (ΔE^P) and average exciton lifetimes $<\tau>$ of the involved molecules in charged-excited state degradation.

Exciplex	material	bond	state	$<\tau_S>$ (ns)	$<\tau_T>$ (ns)	ΔE_S^P (eV)	ΔE_T^P (eV)	ΔE_{red}^P (eV)
TCTA: T2T	TCTA	C-N (A)	cationic	231	273	2.74	2.78	3.17
			anionic			-0.46	-0.42	-0.03
		C-N (B)	cationic			1.65	1.69	2.08
			anionic			-0.85	-0.81	-0.42
	T2T	C-C (A)	cationic			3.53	3.57	3.96
			anionic			2.59	2.63	3.02
		C-C (B)	cationic			3.75	3.79	4.18
			anionic			3.21	3.25	3.64

NPB: T2T	NPB	C-N (A)	cationic	30	56	2.78	2.81	3.16
			anionic			0.02	0.05	0.40
		C-C (B)	cationic			4.83	4.86	5.21
			anionic			2.44	2.47	2.82
		C-N (C)	cationic			1.50	1.53	1.88
			anionic			-0.32	-0.29	0.06
		C-C (D)	cationic			1.66	1.69	2.04
			anionic			-0.27	-0.24	0.11
	T2T	C-C (A)	cationic			3.58	3.61	3.96
			anionic			2.64	2.67	3.02
		C-C (B)	cation			3.80	3.83	4.18
			anionic			3.26	3.29	3.64
NPB: SpiroCO-mTRZ	NPB	C-N (A)	cationic	51	85	2.86	2.89	3.16
			anionic			0.10	0.13	0.40
		C-C (B)	cationic			4.91	4.94	5.21
			anionic			2.52	2.55	2.82
		C-N (C)	cationic			1.58	1.61	1.88
			anionic			-0.24	-0.21	0.06
		C-C (D)	cationic			1.74	1.77	2.04
			anionic			-0.19	-0.16	0.11
	SpiroCO-mTRZ	C-C (A)	cationic			4.27	4.30	4.57
			anionic			3.21	3.24	3.51
		C-N (B)	cationic			1.34	1.37	1.64
			anionic			0.27	0.30	0.57
		C-C (C)	cationic			1.10	1.13	1.40
			anionic			0.99	1.02	1.29
NPB: SF2-TRZ	NPB	C-N (A)	cationic	63	114	2.82	2.85	3.16
			anionic			0.06	0.09	0.40
		C-C (B)	cationic			4.87	4.90	5.21
			anionic			2.48	2.51	2.82
		C-N (C)	cationic			1.54	1.57	1.88
			anionic			-0.28	-0.25	0.06
		C-C (D)	cationic			1.70	1.73	2.04
			anionic			-0.23	-0.20	0.11
	SF2-TRZ	C-C (A)	cationic			3.98	4.01	4.32
			anionic			2.83	2.86	3.17
		C-C (B)	cationic			3.48	3.51	3.82
			anionic			3.33	3.36	3.67
		C-C (C)	cationic			3.51	3.54	3.85
			anionic			2.76	2.79	3.10

BCzPh: SF2-TRZ	BCzPh	C-N (A)	cationic	64	117	1.61	1.64	2.17
			anionic			-1.13	-1.10	-0.57
		C-C (B)	cationic			4.21	4.24	4.77
			anionic			1.26	1.29	1.82
	SF2-TRZ	C-C (A)	cationic			3.76	3.79	4.32
			anionic			2.61	2.64	3.17
		C-C (B)	cationic			3.26	3.29	3.82
			anionic			3.11	3.14	3.67
		C-C (C)	cationic			3.29	3.32	3.85
			anionic			2.54	2.57	3.10
BCzPh: T2T	BCzPh	C-N (A)	cationic	966	1216	1.70	1.72	2.17
			anionic			-1.04	-1.02	-0.57
		C-C (B)	cationic			4.30	4.32	4.77
			anionic			1.35	1.37	1.82
	T2T	C-C (A)	cationic			3.49	3.51	3.96
			anionic			2.55	2.57	3.02
		C-C (B)	cationic			3.71	3.73	4.18
			anionic			3.17	3.19	3.64

Table S7. Calculated bond dissociated energies (ΔE_{TTA}) of the involved molecules in highly excited state degradation.

Material	bond (position)	average BDE (eV)	ΔE_{TTA}
TCTA	C-N (A)	3.95	-0.19
	C-N (B)	3.67	-0.47
T2T	C-C (A)	5.19	1.05
	C-C (B)	5.20	1.06
NPB	C-N (A)	3.75	-0.39
	C-C (B)	5.33	1.19
	C-N (C)	3.51	-0.63
	C-C (D)	3.66	-0.48
SpiroCO-mTRZ	C-C (A)	5.19	1.05
	C-N (B)	3.34	-0.80
	C-C (C)	3.50	-0.64
BCzPh	C-N (A)	3.87	-0.27
	C-C (B)	4.79	0.65

SF2-TRZ	C-C (A)	5.22	1.08
	C-C (B)	5.18	1.04
	C-C (C)	5.17	1.03

Table S8. Electroluminescence performance of the phosphorescent OLEDs using exciplex cohosts in various HTM: ETM ratios.

Ratio [a]	V_{ON} [b] (V)	Voltage/ CE/ EQE (V/ cd A^{-1}/ %) Maximum	at 1000 cd m^{-2}	at 10000 cd m^{-2}	CIE (x, y) [c]
6% PQ2Ir(dpm): NPB: T2T					
3:7	2.2	32.0/ 18.0	3.6/ 32.0/ 18.0	5.4/ 28.9/ 16.1	(0.61, 0.39)
4:6	2.2	33.5/ 18.5	3.4/ 33.2/ 18.4	5.5/ 31.0/ 17.1	(0.61, 0.39)
5:5	2.2	31.6/ 17.2	3.5/ 31.0/ 17.0	5.5/ 29.6/ 16.1	(0.61, 0.39)
6% PQ2Ir(dpm): NPB: Spiro-mTRZ					
4:6	2.1	31.1/ 17.2	3.4/ 31.0/ 17.2	5.2/ 28.4/ 15.6	(0.61, 0.39)
6:4	2.1	32.7/ 17.7	3.3/ 32.6/ 17.7	5.0/ 28.9/ 15.5	(0.61, 0.39)
7:3	2.2	32.8/ 17.6	3.4/ 32.8/ 17.6	5.2/ 28.2/ 14.9	(0.61, 0.39)
6% PQ2Ir(dpm): NPB: SF2-TRZ					
3:7	2.4	28.0/ 15.8	3.8/ 27.7/ 15.6	5.8/ 26.4/ 14.9	(0.61, 0.39)
5:5	2.2	29.9/ 16.7	3.6/ 29.8/ 16.6	5.4/ 27.7/ 16.0	(0.61, 0.39)
6:4	2.2	29.2/ 15.7	3.3/ 28.6/ 15.5	5.1/ 27.1/ 14.5	(0.61, 0.39)
8:2	2.2	28.6/ 15.1	3.5/ 28.4/ 15.0	5.6/ 25.7/ 13.5	(0.61, 0.39)
6% PQ2Ir(dpm): BCzPh: T2T					
4:6	2.3	34.0/ 20.4	3.6/ 33.5/ 20.0	5.7/ 29.7/ 17.6	(0.62, 0.38)
5:5	2.4	33.6/ 19.7	3.7/ 33.3/ 19.5	5.7/ 30.0/ 17.5	(0.62, 0.38)
6:4	2.4	35.7/ 20.7	3.8/ 35.7/ 20.7	5.8/ 32.6/ 18.7	(0.61, 0.38)
7:3	2.4	36.0/ 20.6	3.6/ 35.7/ 20.5	5.6/ 32.4/ 18.5	(0.61, 0.38)
6% PQ2Ir(dpm): BCzPh: SF2-TRZ					
4:6	2.3	33.6/ 19.8	3.7/ 33.2/ 19.6	5.9/ 29.0/ 17.0	(0.62, 0.38)
6:4	2.3	33.7/ 19.9	3.7/ 33.6/ 19.7	5.8/ 30.2/ 17.6	(0.62, 0.38)
7:3	2.3	36.0/ 20.9	3.5/ 35.6/ 20.6	5.5/ 31.6/ 18.2	(0.61, 0.38)
6% PQ2Ir(dpm): TCTA: T2T					
4:6	2.4	35.5/ 19.6	3.5/ 34.0/ 18.6	5.2/ 27.9/ 15.1	(0.60, 0.39)

[a] The ratio is corresponding to proportion of HTM and ETM;

[b] V_{ON} is obtained at 1 cd m^{-2};

[c] CIE (x, y) is obtained at 1 mA cm^{-2}.

Table S9. Operational stability of exciplex cohost OLEDs with various HTM: ETM ratios.

6% PQ2Ir(dpm): NPB: T2T					
NPB: T2T ratio	3:7		4:6		5:5
LT50 (@5000 cd m^{-2}, h)	401.0		689.2		444.1
LT50 (@1000 cd m^{-2}, h) [a]	5266		9051		5832
6% PQ2Ir(dpm): NPB: SpiroCO-mTRZ					
NPB: SpiroCO-mTRZ ratio	4:6		6:4		7:3
LT50 (@5000 cd m^{-2}, h)	274.1		194.3		155.6
LT50 (@1000 cd m^{-2}, h) [a]	3600		2552		2043
6% PQ2Ir(dpm): NPB: SF2-TRZ					
NPB: SF2-TRZ ratio	3:7		4:6		5:5
LT50 (@5000 cd m^{-2}, h)	607.3		423.9		168.6
LT50 (@1000 cd m^{-2}, h) [a]	7975		5567		2214
6% PQ2Ir(dpm): BCzPh: T2T					
BCzPh: T2T ratio	4:6		6:4		7:3
LT50 (@5000 cd m^{-2}, h)	249.5		483.4		638.2
LT50 (@1000 cd m^{-2}, h) [a]	3277		6348		8381
6% PQ2Ir(dpm): BCzPh: SF2-TRZ					
BCzPh: SF2-TRZ ratio	3:7	4:6	5:5	6:4	7:3
LT50 (@5000 cd m^{-2}, h)	516.4	774.3	493.2	350.1	163.7
LT50 (@1000 cd m^{-2}, h) [a]	6782	10169	6477	4598	2150

Table S10. TrEL decay changes and relative exciton quenching rates (v_Q) in the pristine and degraded OLEDs using exciplex cohost.

Exciplex host	state	χ^2	τ_1 (μs)	A1 (%)	τ_2 (μs)	A2 (%)	area ($\times 10^6$)	ΔA (%)	v_Q [a] ($\%\cdot h^{-1}$)
TCTA: T2T	pristine	0.65	0.70	39.6	1.05	60.4	2.78	-5.43	1.51×10^{-2}
	T60	0.87	0.76	87.1	1.47	12.9	2.63		
NPB: T2T	pristine	0.86	0.62	54.3	1.10	45.7	4.26	-13.91	1.54×10^{-3}
	T50	0.76	0.57	54.1	0.99	45.9	3.67		
NPB: SpiroCO-mTRZ	pristine	0.80	0.61	55.2	1.06	44.8	4.03	-11.31	3.14×10^{-3}
	T50	0.73	0.56	59.1	1.02	40.9	3.58		
NPB: SF2-TRZ	pristine	0.94	0.68	67.7	1.27	32.3	4.24	-8.66	1.09×10^{-3}
	T50	0.93	0.70	86.3	1.59	13.7	3.87		
BCzPh: T2T	pristine	0.61	0.85	88.9	1.95	11.1	4.57	-3.23	3.86×10^{-4}
	T50	0.93	0.84	87.3	3.57	12.7	4.43		
BCzPh: SF2-TRZ	pristine	0.78	0.72	59.8	1.11	40.2	4.44	-6.37	6.27×10^{-4}
	T50	1.23	0.78	89.4	2.36	10.6	4.15		

[a] Relative exciton quenching rate v_Q can be calculated as $v_Q = |\Delta A|/$ device lifetime.

Table S11. Carrier injection changes and the relative carrier trap generation rate (v_{CT}) in the pristine and degraded devices.

Exciplex host	A_{Fresh} (nF·V)	$A_{Degraded}$ (nF·V)	ΔA [a] (%)	Device lifetime (h)	v_{CT} [b] ($\%\ h^{-1}$)
TCTA: T2T (4:6)	0.637	0.388	-39.2	380	1.03×10^{-1}
NPB: T2T (4:6)	0.596	0.514	-13.8	9051	1.52×10^{-3}
NPB: SpiroCO-mTRZ (4:6)	0.618	0.538	-12.9	3600	3.58×10^{-3}
NPB: SF2-TRZ (3:7)	0.298	0.204	-31.4	7975	3.93×10^{-3}
BCzPh: T2T (7:3)	0.730	0.329	-55.0	8381	6.56×10^{-3}
BCzPh: SF2-TRZ (3:7)	0.368	0.240	-34.9	6782	5.15×10^{-3}
BCzPh: SF2-TRZ (4:6)	0.470	0.215	-54.3	10169	5.34×10^{-3}
BCzPh: SF2-TRZ (5:5)	0.424	0.216	-48.9	6477	7.55×10^{-3}
BCzPh: SF2-TRZ (7:3)	0.511	0.286	-44.0	4598	9.58×10^{-3}

[a] Decay of carrier injection peak area $\Delta A = (A_{Fresh} - A_{Degraded})/A_{fresh}$;

[b] Relative carrier trapping rate $v_{CQ} = |\Delta A|/$ device lifetime.
